# Machine learning in TCM with natural products and molecules: current status and future perspectives

**DOI:** 10.1186/s13020-023-00741-9

**Published:** 2023-04-20

**Authors:** Suya Ma, Jinlei Liu, Wenhua Li, Yongmei Liu, Xiaoshan Hui, Peirong Qu, Zhilin Jiang, Jun Li, Jie Wang

**Affiliations:** 1 Guang’anmen Hospital, China Academy of Chinese Medicine Sciences, Beijing, 100053 China; 2 Tianjin University of Traditional Chinese Medicine, Tianjin, 301617 China

**Keywords:** Machine learning, Deep learning, Traditional Chinese medicine, Natural products, Chemical components, Multidisciplinary intersection

## Abstract

Traditional Chinese medicine (TCM) has been practiced for thousands of years with clinical efficacy. Natural products and their effective agents such as artemisinin and paclitaxel have saved millions of lives worldwide. Artificial intelligence is being increasingly deployed in TCM. By summarizing the principles and processes of deep learning and traditional machine learning algorithms, analyzing the application of machine learning in TCM, reviewing the results of previous studies, this study proposed a promising future perspective based on the combination of machine learning, TCM theory, chemical compositions of natural products, and computational simulations based on molecules and chemical compositions. In the first place, machine learning will be utilized in the effective chemical components of natural products to target the pathological molecules of the disease which could achieve the purpose of screening the natural products on the basis of the pathological mechanisms they target. In this approach, computational simulations will be used for processing the data for effective chemical components, generating datasets for analyzing features. In the next step, machine learning will be used to analyze the datasets on the basis of TCM theories such as the superposition of syndrome elements. Finally, interdisciplinary natural product-syndrome research will be established by unifying the results of the two steps outlined above, potentially realizing an intelligent artificial intelligence diagnosis and treatment model based on the effective chemical components of natural products under the guidance of TCM theory. This perspective outlines an innovative application of machine learning in the clinical practice of TCM based on the investigation of chemical molecules under the guidance of TCM theory.

## Introduction

Machine learning, which involves learning relationships from data using computer science, has been successfully applied to solve complicated tasks such as computer vision, speech recognition, and natural language processing [1, 2]. The large amount of TCM data which utilized natural product (herbal medicine) to remedy disease produced from long-term clinical diagnoses, treatment and experiments, can be utilized for future research with machine learning. Machine learning verifies similarities among datasets by identifying characteristic regularities between input data and output results, and it has been applied for research on natural products, disease diagnosis and treatment, etc. [3]. Deep learning is an extension of machine learning that involves processing and validation of large training datasets between input and output units [4]. Deep learning has gained increasing importance for effective processing of large amounts of data and identifying patterns or functions hidden deep inside biological data. It has rapidly developed and successfully applied in many fields, including image recognition, robotics, speech recognition, and life sciences [5]. Deep learning uses different structural network models for different types of data and different application situations, and the primary models in deep learning include the convolutional neural network (CNN), Elman recurrent neural network (RNN), long short-term memory (LSTM), and generative adversarial network (GAN). Unlike CNN, RNN, and LSTM, which are deep neural network models, GAN is an unsupervised learning algorithm that learns by playing two deep neural networks against each other.

TCM is one of the oldest healthcare systems in the world and is being increasingly used as a complementary medicine system worldwide [6]. As a fully institutionalized part of Chinese healthcare, TCM is widely used with western medicine in China. Natural products which contain various chemical ingredients are employed to cure disease under the guidance of TCM theory. TCM theories such as the eight diagnostic principles to differentiate, the five elements theory, and the visceral manifestation theory can be collected by four traditional examination methods: looking, listening and smelling, asking, and touching, which could obtain pulse, face, tongue, urine, and stool information to provide essential information for diagnosis and natural products treatment. The process of diagnosis that guides treatment is called syndrome differentiation, which reflects the temporary state of a syndrome defined on the basis of the symptoms and signs identified by the four traditional examination methods. In this approach, wherein a clinical condition defined as a specific disease in western medicine can manifest in different syndrome elements in the same patient and may require varying treatment over time [7]. When this guiding TCM theory could be reproduced by machine learning, natural products which treat disease basing on chemical ingredients and molecules will be more powerful in remedying disease, that will bring major changes to human health and the quality of human life and save more lives. Although machine learning in TCM have been studied before [8–10], in the present study, we provide a systemic summary both of machine learning and its application in TCM, and proposed a promising future research direction. We summarized the principles and processes underlying deep learning and traditional machine learning algorithms, analyzed the development and application of machine learning in TCM research, and proposed a promising research direction that integrates machine learning with TCM theory, natural products research, and computational simulation to provide an intelligent artificial intelligence diagnosis and treatment model based on the effective chemical components of natural products and molecules under the guidance of TCM theory.

## Machine learning algorithms-deep learning

### Convolutional neural network

**Fig. 1:**
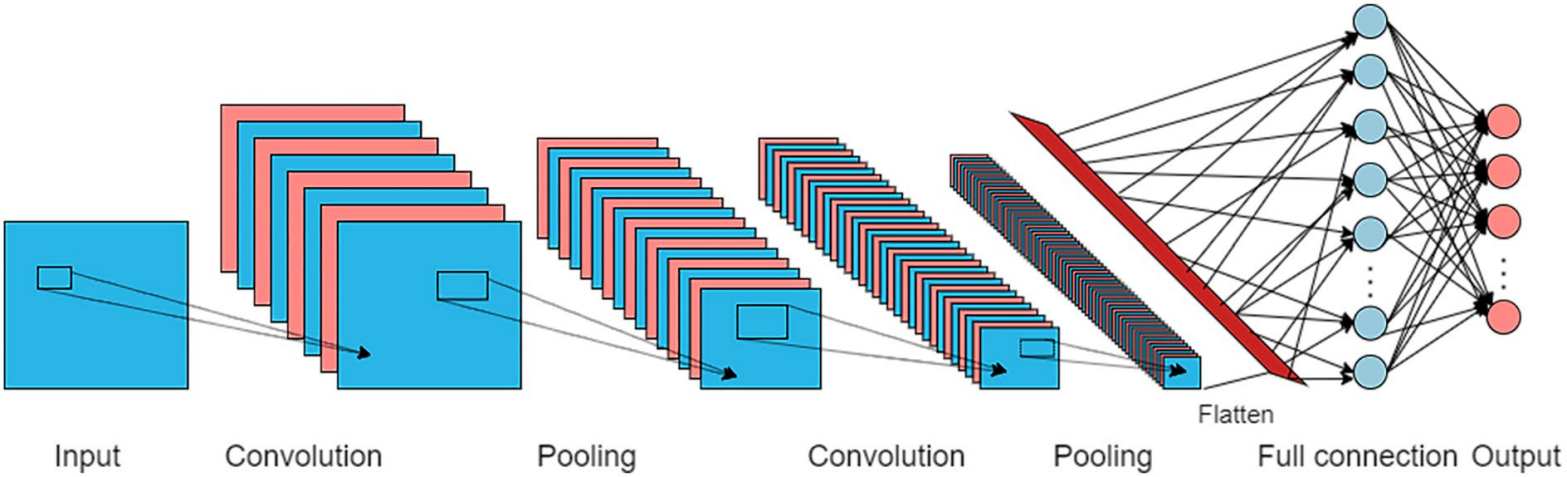
The basic structure of CNN, including input, convolution, pooling, full connection and output layers

CNN is a deep feedforward neural network with the characteristics of local connection and weight-sharing that uses a stack of convolution layers to extract features. It is widely used in image classification [11], facial recognition [12], semantic segmentation [13], object detection [14], and natural language processing [15]. The core idea of CNN involves a local receptive field, weight-sharing, and a pooling layer. The architecture of CNN consists of a sequence of layers that function as follows: when data is input into the CNN, the convolution layer extracts the features and the pooling layer aggregates the local features extracted by the convolution layer to obtain global features. Finally, the fully connected layer is combined to classify and output the results (Fig. 1). The convolution kernel (also named as filter) is utilized in the convolution layer with sizes of 1 × 1, 3 × 3, or 5 × 5. Activation is applied after each convolution layer. The ReLU function, a mathematical formula that chooses the maximum of either z or 0 and is designated as $f\left(z\right)={max}(0,z)$, is often utilized. Then, the pooling layer is used to reduce location sensitivity, minimize the number of parameters and computation in the network, and to control overfitting [16]. The most common pooling function is the MAX pooling function, which uses the maximum value from each cluster of neurons at the prior layer to form a new neuron in the next layer. Other functions such as average pooling are also applicable [16]. The pooling layer reduces the input dimension of the subsequent network layer, reduces the size of the model, improves the calculation speed, improves the robustness of the feature map, and prevents overfitting. Three hyperparameters­depth, stride, and padding are used to control the size of the output data volume. The depth is consistent with the number of filters used, while the stride parameter reflects the number of pixels by which the filter moves each time it slides. The filling layer reflects the filling at the edge of the data volume, which can be filled with 0 or the mean value. CNN minimizes losses by adjusting the network parameters iteratively, and improves the accuracy of the network through frequent iterative training [17].

### Elman recurrent neural network

**Fig. 2:**
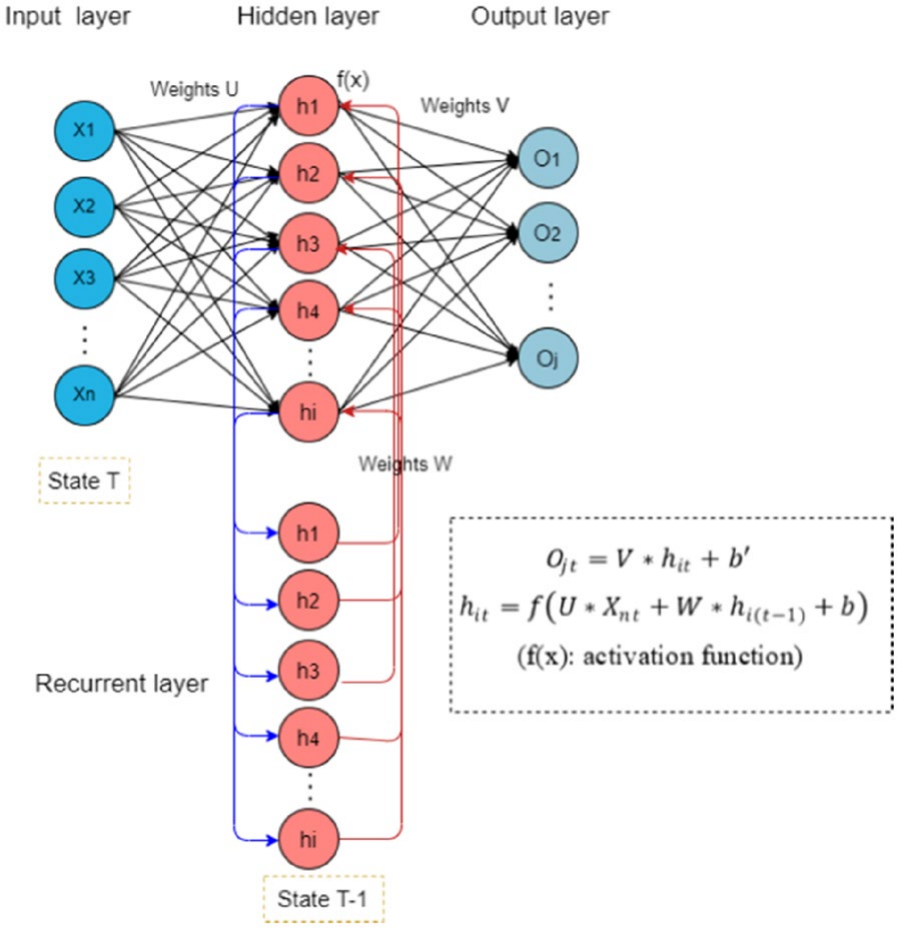
The basic structure of Elman RNN, consists of input, recurrent, hidden, and output layers. *U*, *V* and *W* are the weights of input layer, output layer, recurrent layer separately. Parameter *b* represents bias term of hidden layer, *b’* represents bias term of output layer. Hidden layer: ${h}_{it}=f(U*{X}_{nt}+W*{h}_{i\left(t-1\right)}+b)$. Output layer:${O}_{jt}=V*{h}_{it}+b{\prime }$

The Elman RNN, also named as a simple recurrent network, was the first RNN among feedback neural networks and was specifically designed for processing time-dependent sequential data. The Elman RNN is often used in natural language processing [18]. It shows both current and past features of time series, adapts to the long-term historical changes in data, stores past information to solve context-dependent tasks, and provides predictions simultaneously with existing observations [2]. The Elman RNN consists of input, recurrent, hidden, and output layers. The standard connections of each layer which are similar to a feedforward network, are applied synchronously to propagate information from one layer to another by calculating a nonlinear function [19]. The input layer plays a signal transmission role, and the output layer plays a weighting role. The hidden and output layers usually employ the sigmoid nonlinear function as the activation function [20]. The recurrent layer is utilized to memorize the output value of the hidden layer at the previous moment, which can be regarded as a one-step delay operator. By the recurrent layer, the output of the hidden layer can self-connected to the input through delay and storage facilitated, and this self-connection makes the network can capture historical information. The addition of the internal feedback network increases the capacity of the network to handle dynamic information, thereby allowing dynamic modeling [21] (Fig. 2).

### Long short-term memory network

**Fig. 3:**
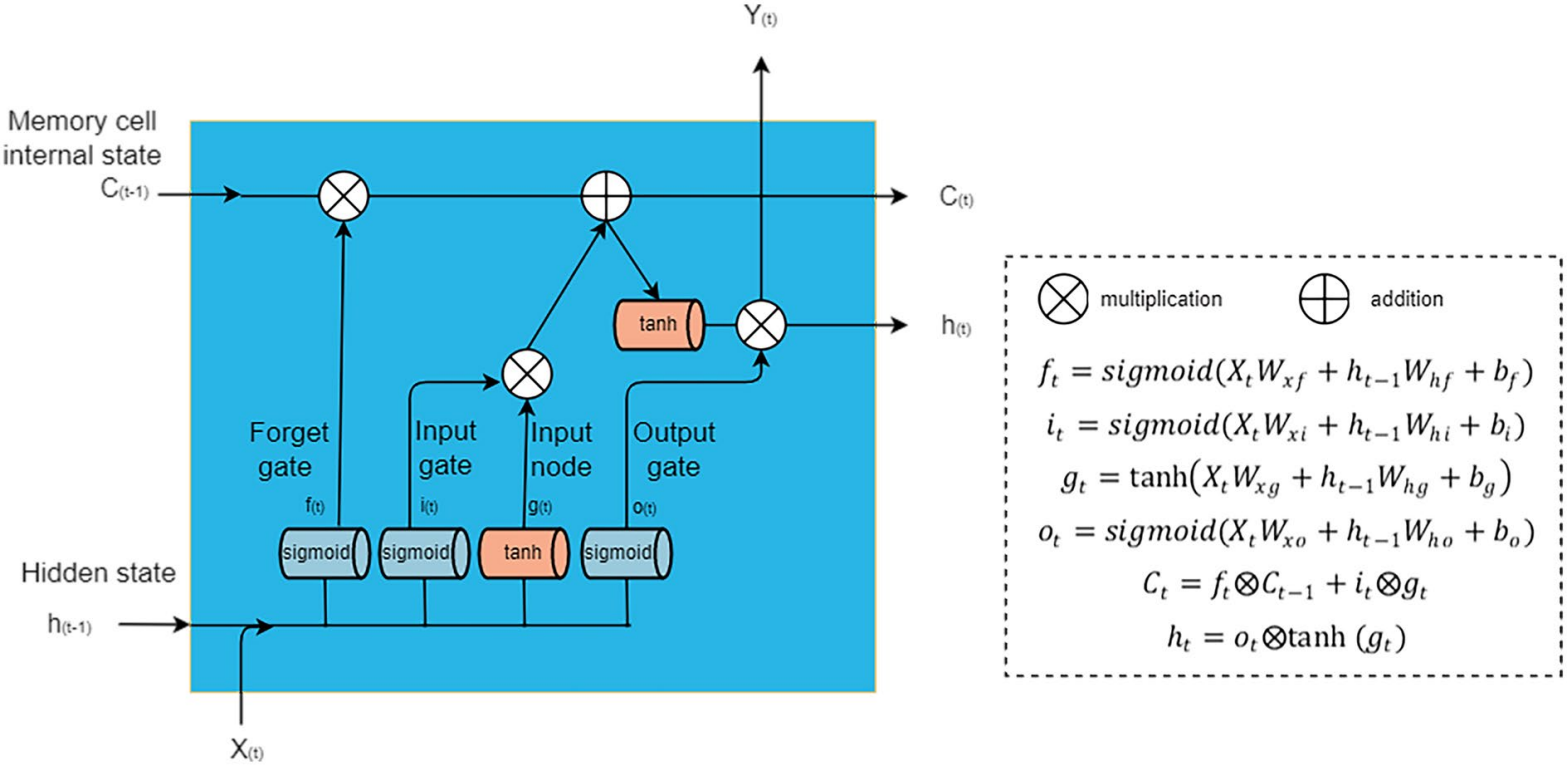
The basic structure of LSTM. Input *X(t)*, output *Y(t)*; *W* represents weight, *b* represents bias term. Hidden state: ${h}_{t}={o}_{t}\otimes \text{t}\text{a}\text{n}\text{h}\left({g}_{t}\right)$; input node: ${g}_{t}=\text{t}\text{a}\text{n}\text{h}({X}_{t}{W}_{xg}+{h}_{t-1}{W}_{hg}+{b}_{g})$; memory cell internal state: ${C}_{t}= {f}_{t}\otimes {C}_{t-1}+{i}_{t}\otimes {g}_{t}$. Input gate: ${i}_{t}=sigmoid({X}_{t}{W}_{xi}+{h}_{t-1}{W}_{hi}+{b}_{i})$; forget gate: ${f}_{t}=sigmoid({X}_{t}{W}_{xf}+{h}_{t-1}{W}_{hf}+{b}_{f})$; output gate: ${o}_{t}=sigmoid({X}_{t}{W}_{xo}+{h}_{t-1}{W}_{ho}+{b}_{o})$

The LSTM is an advanced variant of RNN with the capability of preserving long-term dependencies by using internal feedback [22]. Essentially, the LSTM layers prevent older information from gradually vanishing [23]. The LSTM is a popular RNN and has been successfully applied in many fields such as speech recognition, image description, and natural language processing. The LSTM can make use of gating mechanisms to mitigate gradient exploding and gradient vanishing when learning long-term dependencies [24]. This model introduces an intermediate type of storage using memory cells. A memory cell is a composite unit built from simpler nodes in a specific connectivity pattern, with the novel inclusion of multiplicative nodes. Each memory cell is equipped with an internal state and a number of multiplicative gates, namely, the input, forget, and output gates. The input gate determines whether a given input should impact the internal state; the forget gate determines the extent to which the internal state should be flushed; and the output gate determines the extent to which the internal state of a given neuron should be allowed to influence the cell’s output. The LSTM uses two activation functions: the tanh function and the sigmoid function. The repetitive module of the Elman RNN contains only one tanh function, while the repetitive module in the LSTM contains four interacting activation functions (three sigmoid and one tanh) (Fig. 3).

### Generative adversarial network

**Fig. 4:**
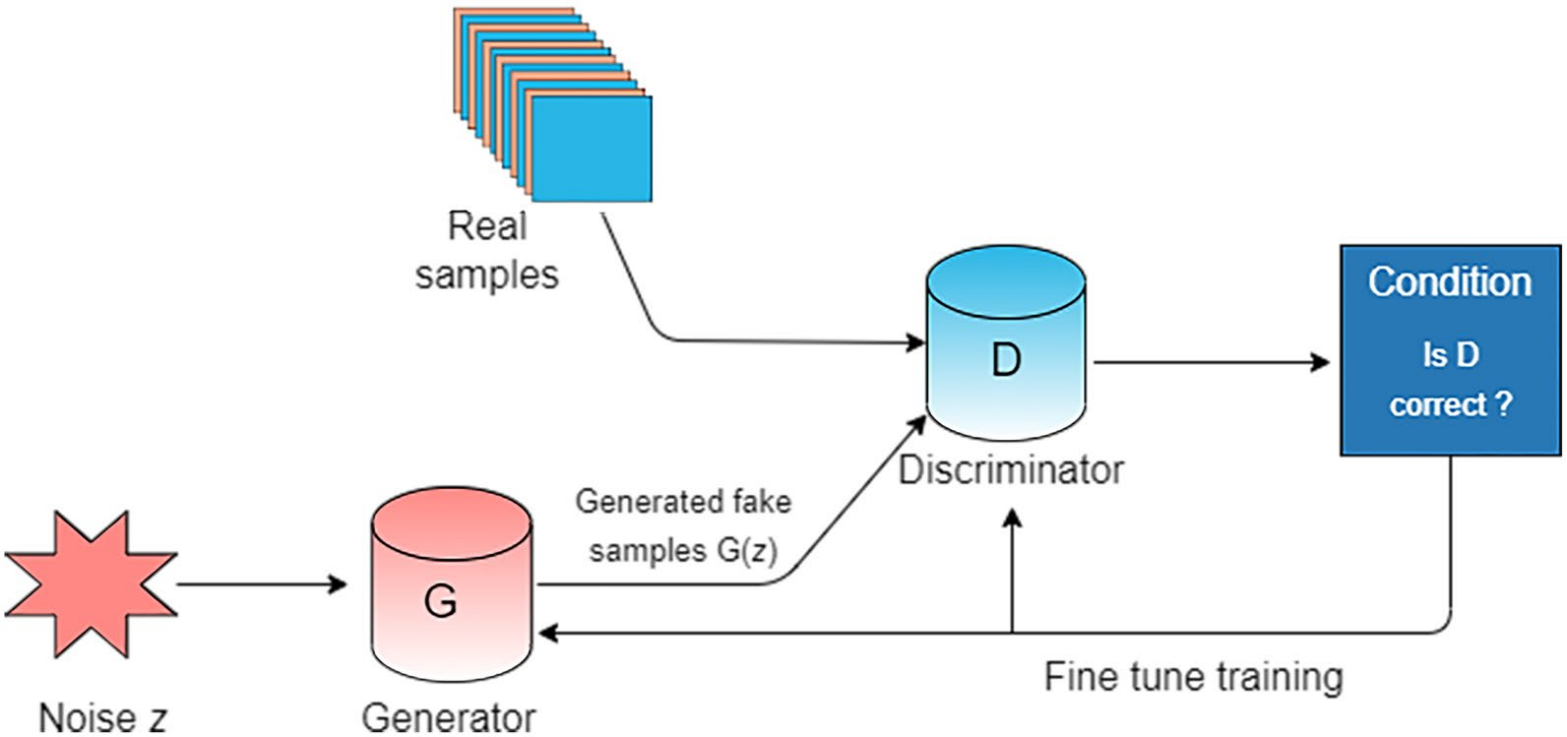
The basic structure of GAN structure, consisting of a generator and a discriminator

GAN is a promising framework composed of two components: a generator and a discriminator. The generator generates false data samples and tries to deceive the discriminator. The discriminator tries to distinguish between true and false samples, which compete with each other in the training phase [25]. By repeating these steps, the generator and discriminator continue to improve in their respective tasks (Fig. 4). The generator and discriminator are usually implemented by a neural network with simultaneous training, and both of them are trained by playing minimax games from game theory. The generator maximizes the cross-entropy loss (i.e., $\text{max}\text{l}\text{o}\text{g}\left(D\right(x{\prime }\left)\right)$), while the discriminator minimizes the cross-entropy loss (i.e., $min-ylogD\left(x\right)-(1-y)\text{l}\text{o}\text{g}(1-D(x\left)\right)$). The adversarial loss created by the discriminator provides a clever approach to incorporate unlabeled samples into training and impose higher-order consistency. This model has achieved state-of-the-art performance in many image-generation tasks, including text-to-image synthesis, super-resolution, and image-to-image translation [26].

## Machine learning algorithms-traditional machine learning algorithms

### Multilayer perceptron

**Fig. 5:**
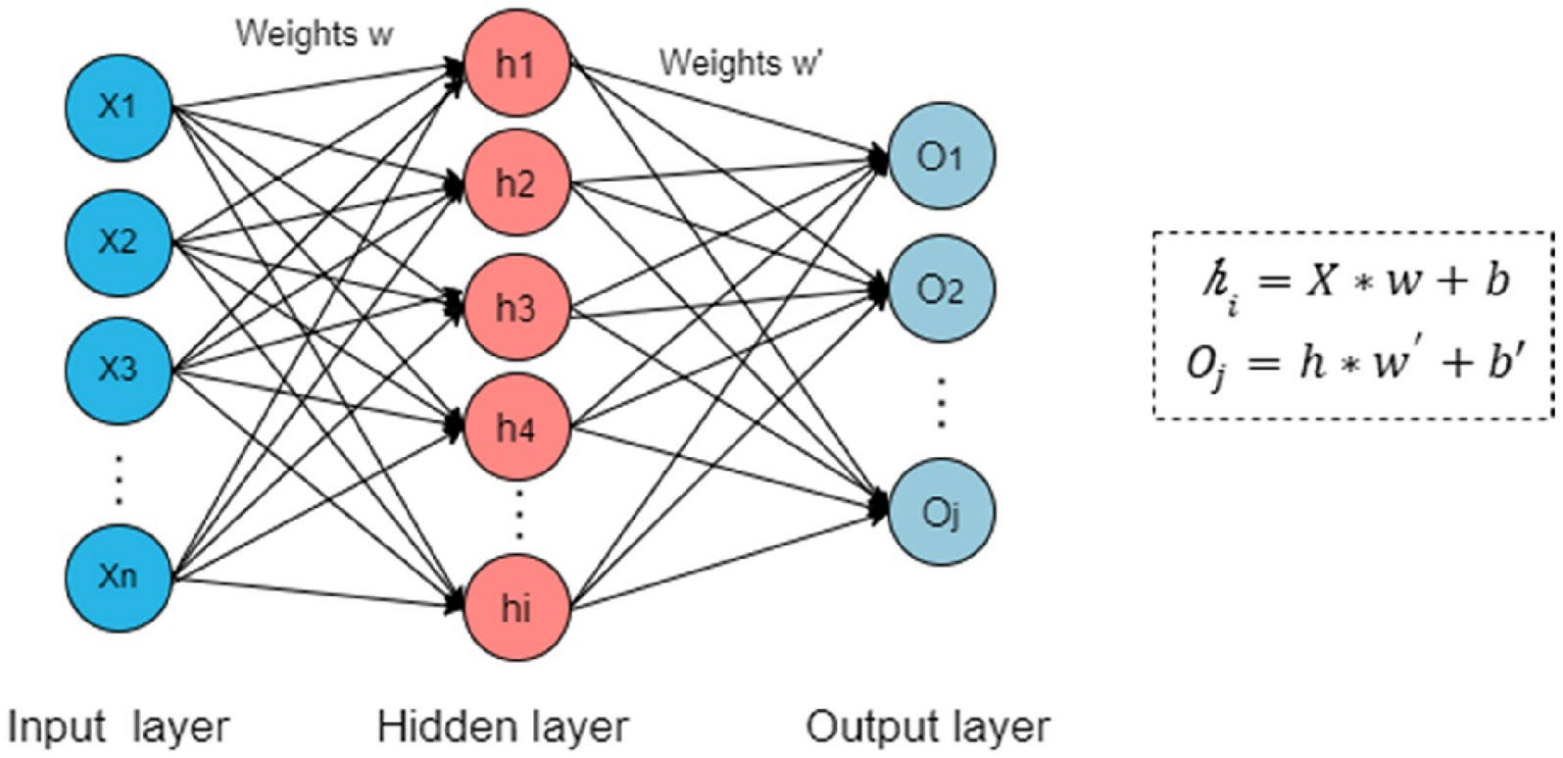
The basic structure of MLP, including input layer, hidden layer, and output layer. The parameter *w* and *w’* represent weight, *b* and *b’* represent bias term. Hidden layer: ${h}_{i}=X*w+b$; output layer: ${O}_{j}=h*{w}^{{\prime }}+b{\prime }$

Multilayer perceptron (MLP) uses a neural network with fully connected layers in a nonlinear model, including an input layer, several hidden layers, and an output layer with the capability to calculate the weighted sum of its inputs and then apply an activation function to transform a signal to the next neuron [27] (Fig. 5). The ReLU, sigmoid, and tanh functions are the common activation functions in the MLP. The sigmoid function, which takes a real-value input and “squashes” it in a range between 0 and 1, was often used previously. Like the sigmoid function, the tanh function also squashes its inputs, transforming them into elements on the interval between − 1 and 1. However, the ReLU function has now emerged as a more popular nonlinear function with a mathematical formula that chooses the maximum of either x or 0 [16]. The ReLU function is significantly more amenable to optimization than the sigmoid or the tanh function. MLP utilizes the back-propagation method with stochastic gradient descent to training [28].

### Support vector machine, decision tree, random forest

The support vector machine (SVM) algorithm is a generalized linear classifier model for binary classification [29]. By using a non-parametric max-margin classification technique, it can classify data into two groups [30]. However, since practical research usually involves nonlinear problems, high-dimensional linear separable problems should be used instead of low-dimensional linear inseparable problems. Kernel functions such as the Gaussian, linear, and polynomial kernel functions are used to address this issue. SVM is based on the principle of structural risk minimization and shows excellent characteristics in nonlinear and small-sample problems. Combinations of methods or modification of separating hyperplanes, classification margins, boundaries, etc., can improve the generalization of SVM [30], avoiding the under-fitting and overfitting problems in previous attempts at neural network learning and yielding high generalization ability [31]. Decision tree (DT) is a basic classification and regression method in machine learning that mainly includes feature selection, decision tree generation, and pruning [32, 33]. Common DT algorithm models include the ID3, C4.5, and CART algorithms [34]. The DT algorithm is simple and intuitive, easy to understand, shows enough flexibility and expression ability. The random forest (RF) algorithm is an extension of DT with a high-performance speed [35, 36]. RF can perform prioritization of features by assigning different weight coefficients to different categories [37]. RF works by sequentially injecting training data and feature vectors into each of the base learners, identification of the best subset of features, and achieving the highest performance among all the aggregated base learners by increasing the impact factor of the best-feature subset in the classifier [38].

### Comparisons between algorithms

From the original AlexNet in 2012 through the VGG in 2014 and ResNet in 2015, CNNs have been predominantly used in the field of computer vision [39] and natural language processing [40]. CNNs show good fitting effect and high accuracy. The weight-sharing feature of CNNs reduces the number of parameters, while their shift-invariant feature enhances the robustness of the network and shows an anti-disturbance effect. However, the shift-invariant feature also means that slight changes in the object will not activate the recognition of the object neurons. Moreover, the CNN model pooling layer loses a lot of valuable information, and the lack of a memory function as well as the limited data size and high computational requirements are other shortcomings of CNN [41] (Table 1). The Elman RNN has shown good ability in capturing the dynamics of sequences via recurrent connection, e.g., as in natural language processing [22]. The Elman RNN is effective and shows good generalization ability and has been widely used for solving practical problems [21]. However, it is prone to show gradient explosion and gradient vanishing, and cannot address the problems of long-term dependencies and parallel training [42]. In comparison with the Elman RNN, LSTM can achieve better analysis results in longer sequences, solving the vanishing gradient problem and stability problems in the time dimension of Elman RNN. However, the use of LSTM for processing longer sequence data is still difficult. Moreover, its calculation is time-consuming [22] (Table 1). As a generative model, GAN only uses back-propagation, which improves efficiency. GAN is an unsupervised learning method and is good at generalization. It can be used when the probability density cannot be calculated. The main disadvantage of GAN is the unstable training process and the difficulty in achieving Nash equilibrium. GAN can be used for various learning tasks, especially in the field of computer vision, but it is not suitable for processing discrete forms of data, such as text [25] (Table 1).

MLP is a simple and easy-to-implement algorithm with good generalization ability that is often used for identification, classification, and prediction [32]. However, there are two main problems associated with the development of MLP networks: architecture optimization and training. The definition of the architecture is a critical point because the lack of connections can reduce the ability of the network to solve the problem of insufficient adjustable parameters, while too many connections may lead to overfitting of the training data. Therefore, training for large datasets is very time-consuming with MLP [27] (Table 1). SVM is more suitable for binary classification with small sample sizes and shows better robustness and generalization ability [43]. However, SVM is sensitive to parameters and kernel functions, and it is not suitable for multi-classification research in the case of non-optimization. SVM is often used in data classification and regression [32] (Table 1). As common traditional machine learning algorithms, DT and RF are based on simple principles and are easy to implement and can be used for data classification and regression [32, 44]. However, DTs are unstable since small variations in the data may result in the generation of a completely different tree. On the other hand, although RF shows good capability to reduce data noise, it is prone to overfitting when training large amounts of data [45]. RF has a simple structure, and is ease of understanding, performs higher efficiency than similar methods [37] (Table 1).

Deep learning offers absolute advantages in computer vision and natural language processing. Its powerful processing capabilities for features such as image and time series features are beyond the reach of traditional machine learning algorithms. Deep learning methods such as generative adversarial algorithms and reinforcement learning ensure continuous improvements in the calculation accuracy, which is also beyond the reach of traditional machine learning algorithms. However, for small datasets, deep learning is prone to overfitting and shows no advantage over traditional machine learning algorithms. Thus, the development of artificial intelligence techniques for TCM will require a combination of deep learning and traditional machine learning. Deep learning is preferred in feature extraction, such as semantic segmentation data fitting and image feature extraction. In contrast, traditional machine learning algorithms such as MLP and RF may be more suitable for small data classification and regression problems (Table 1).

**Table 1:** Comparisons between machine learning algorithms

Machine learning algorithm		Advantages	Limitations	Applications
Deep learning	CNN	High accuracy; Weight-sharing; Relieves the model overfitting problem; Shift-invariant feature enhances the robustness of the network	The pooling layer loses a lot of valuable information; Substantial hardware and dataset size requirements [41]; No memory function; Shift-invariant feature also prevents the neuron that recognizes the object from being activated when the object changes slightly	Computer vision [40, 41]; Natural language processing [40]
	Elman RNN	Strong ability to extract time series features; Better generalization ability	Prone to show gradient exploding and gradient vanishing; Unable to solve the problem of long-term dependencies and parallel training [42]	Time series data, e.g., natural language processing [22]
	LMST	Achieve better analysis results in longer sequences; Solving vanishing gradient problem and stability problems in the time dimension of Elman RNN	When processing longer sequences data, LSTM is still difficult; Time-consuming [22]	For processing longer time series data than Elman RNN, such as in natural language processing [22]
	GAN	Generative model; Can still be used when the probability density is not calculated; Good at generalization	Unstable training process; Difficult to achieve Nash equilibrium; Not suitable for processing discrete forms of data	Data augmentation [46]; Text-to-image synthesis [26]; Image-to-image translation [26]; Computer vision [25]
Traditional machine learning	MLP	Simple model; Easy implementation, and good generalization ability	architecture optimization; For training large datasets is very time-consuming [27]	Identification; Classification and prediction [32]
	SVM	Suitable for small-sample binary classification research; Good robustness and generalization ability	Sensitive to parameters and kernel function; Inappropriate for multi-classification research in non-optimized cases	Classification and Regression problems [32]
	DT	Simple to understand and to interpret; Requires little data preparation	Unstable for small variations in the data might result in the generation of a completely different tree	classification, and regression problems [32]
	RF	Simple structure; Easy to implement; Higher efficiency [37]	Unable to optimize its own parameters; Overfitting can easily occur when the amount of data is large [45]	Classification and regression problems [44]

## Applications of machine learning in TCM research

### Applications of machine learning in natural products development

Deep learning is widely used in the research and development of natural products (Table 2). Natural products which contain many effective chemical components with great potential value are the main methods to treating diseases in TCM. Approximately 70–95% of people in the developing world continue to rely on natural products as their primary pharmacopeia [47]. Thus, the development of natural products is of a great importance in clinical therapy, especially in combination with machine learning, is an innovative, forward-looking, and applicable new model. The effective scientific characterization of natural products is the basis for using machine learning [48]. Chemical descriptors and fingerprints are often used to quantify the natural products’ effective chemical entities physicochemical characteristics and the related biological target molecules. Chemical descriptors characterized molecules properties by experimental quantification or theoretics which represent its chemical, physical, or topological characteristics. While chemical fingerprints are more complex for encoding as binary bit strings. Chemical fingerprints can reflect the active constituents in substances and can effectively characterize the quality of TCM’s materials [48]. Both molecular descriptors and fingerprints perform crucial functions in machine learning-based applications for drug discovery processes such as target molecule ranking, similarity-based compound search, and virtual screening [49]. For example, machine learning could successfully identify the antibiotic precursor halicin with different structures [50]. The researchers used a deep neural network model that translated the graphical representation of a molecule into a continuous vector through a directed bond-based message passing approach to train the dataset and then used computer simulations to screen compounds that were obtained by vitro screening to finally obtain halicin. Chen [51] used the SVM algorithm to establish a mathematical discriminant model to distinguish the cold and hot nature of natural products. Machine learning can collect and process data based on the medicinal properties, chemical compositions, and function of natural products, allowing automatic discrimination and prediction. Chuang [52] comprehensively discussed how artificial intelligence can address the limitations of molecular descriptors and fingerprints and thereby improve the predictive modeling of compound bioactivities. Yang [53] utilized RF, neural networks, and SVM to identify new compounds in TCM prescriptions for Alzheimer’s disease. They utilized data mining to collect Alzheimer’s disease-related and unrelated compounds from the literature databases. Then, RF, gradient boosting machine and neural networks were utilized to determine the importance of each feature, and important features were selected by molecular descriptors for feature extraction. The selected features were input to the SVM algorithm to identify the new compounds in TCM prescriptions. Yu [54] used RF to obtain the feature descriptors of natural product compounds, SVM to predict hit molecules based on the feature descriptors screened by RF, and molecular docking to perform virtual screening. They successfully identified 4′,5,7-trimethoxyflavone as a potential platelet-derived growth factor receptor α (PDGFRA) inhibitor.

Although the majority of natural products appear inherently safe, clinicians and researchers should also pay attention to the potential for drug-induced injury. The liver which is the major organ of drug metabolism is more likely to show drug-induced injuries than other organs, and these injuries may lead to hepatitis, liver fibrosis, liver failure, and even death [55]. The kidney is also highly susceptible to drug-induced toxic insults that are a common cause of acute kidney injury [56]. With advancements in machine learning, researchers have turned their attention to the use of machine learning applications for evaluating drug-induced injuries. Hu [57] used SVM and in vitro screening to predict and validate the risk of idiosyncratic drug-induced liver injuries caused by the natural products in *Polygonum multiflorum* Thunb, and provided a powerful tool to screen large datasets for toxicants. He [58] established a large-scale dataset focused on TCM-induced hepatoprotection to train machine learning models such as RF and voting models. Their work helped screen potential hepatoprotectants from natural products. Chen [59] developed a method for screening hepatotoxic compounds in TCM and Western medicine combinations on the basis of chemical structures by using SVM, neural networks, DT, and RF. Their results showed that RF yielded a classification accuracy of 0.838, which was better than other machine learning methods.

### Applications of machine learning in disease diagnosis

With the application of AI technology in TCM, AI-assisted disease diagnosis has emerged as a promising research field. With TCM symptoms corresponding to features in the machine learning literature, syndrome elements serve as classes or labels [60], and machine learning has been used in disease diagnosis models (Table 2). Wang [10] used an optimized SVM algorithm to construct a serology-based lung cancer diagnosis model, analyzed the potential therapeutic mechanisms of wogonin in lung cancer, explored the relationship between serological markers and wogonin targets, and constructed a signal pathway regulated by wogonin. Shi [61] developed a new fatigue classification method by integrating pulse data and tongue images with machine learning algorithms and using machine learning models, including SVM, RF, and neural networks, to diagnose disease-related fatigue and non-disease-related fatigue. Senoner [62] achieved good results when using the neural network algorithm with electrocardiogram data to assist the diagnosis of preexcitation syndrome. Using the neural network model based on blood pressure data, Sun [63] established a TCM syndrome diagnosis model of coronary heart disease. Zhang [64] developed a TCM assistive diagnostic system by utilizing bidirectional LSTM with RF for named entity recognition, a CNN for text processing for disease diagnosis, and an integrated learning model for syndrome prediction. Zhao [65] utilized an adaptive resonant neural network for quantitative diagnosis of TCM syndrome types.

Clinical information for TCM diagnosis is collected by the diagnostic methods of looking, listening and smelling, asking, and touching. Intelligent auxiliary diagnosis methods based on these four diagnostic methods in TCM are constantly developing with the accumulation of clinical diagnosis and treatment records, experimental records, TCM databases, books, medical literature, and the other knowledge. Diagnosis based on visual examination is an important method to obtain disease information, and tongue and eye diagnoses are its main components. TCM tongue diagnosis involves interpretation of tongue images obtained by doctors on the basis of the theory of TCM after observing the tongue coating, quality, shape, and other tongue-related information. Tongue diagnosis provides much information about the state of the body, and the diagnostic points of tongue diagnosis include the color and state of the tongue coating, color, texture, shape, and characteristics of the sublingual vein and tongue body parts, etc., which are important features in tongue diagnosis data collection. Traditional machine learning algorithms such as SVM [66], DT, neural networks [45], and RF [67] have been previously used as intelligent auxiliary diagnosis algorithms for tongue diagnosis. The accuracy of SVM in processing hyperspectral red-green-blue tongue images based on tissue type combination can be as high as 93.11% [66]. Liu [68] selected 22 kinds of tongue features in 311 participants to establish the training data set, and used DT (accuracy rate, 66.9%) and MLP (accuracy rate, 64.3%) to classify the tongue images corresponding to kidney deficiency. Qi [67] used the open-source Weka software to classify the color of 728 tongue images, and obtained an RF prediction accuracy of 84.94%. Yan [69] used deep learning and RF to classify normal, mild, and severe teeth-marked tongues. Lu [70] utilized Ridge-CNN to classify sublingual varices of TCM with an accuracy rate of 87.5%.

Although SVM, RF, and MLP have been shown to be effective for simple image classification, they are not satisfactory for complex tasks. With advancements in machine learning, deep learning is being gradually applied for complex task processing for intelligent tongue diagnosis. CNN models are good at image classification, and have been shown to be better than other traditional algorithms in this field [71]. The AlexNet, GoogLeNet, ResNet, and DenseNet network structures with CNN as the model algorithm have been applied for tongue image classification. Huo [72] used a CNN model with an AlexNet network structure and achieved higher accuracy for tongue shape classification as well as reduced training time for the CNN model. Xiao [73] used the improved AlexNet network structure to build a tongue coating color classification model. Using the GoogLeNet network, Christian [74] proposed the inception module to optimize training from another perspective, extracting more features with the same amount of computation. With advancements in CNN, ResNet solved the problem of difficult training, high error rates, and a rapid decline in accuracy after the CNN depth increases. Shao [75] first separated the tongue and tongue coating, and then used the separated images as input to classify the tongue and tongue coating using ResNet-50. Residual connections make the CNN deeper, stronger, and more efficient. DenseNet further expands network connectivity to ensure maximum information flow between layers. Using the AlexNet network structure, Chen [76] introduced the dense connection method in DenseNet and proposed the tongue-coating classification model TonNet.

Eye evaluations can realize intelligent auxiliary diagnosis through fundus image analysis. Retinal vessels are the only visible blood vessels that can be evaluated by simple fundus photography, and this approach provides a convenient method to evaluate cardiovascular status. One study found that retinal features were associated with stroke, and the researchers used the CNN model of the ResNet50 network structure to conduct a stroke risk assessment using retinal images [77]. Sun [78] used CNN to extract features and identify syndromes of yin deficiency, and achieved good results.

Traditional auscultation mainly involves listening to sounds. With advancements in medical treatment, auscultation using equipment is now also utilized in TCM. The combination of an electronic stethoscope with artificial intelligence technology can allow digital acquisition of heart sounds, providing an objective basis for heart sound auscultation. Traditional machine learning methods for heart sound auscultation usually involve segmentation, feature extraction, and classification. Although traditional machine learning methods allows rapid model training, they usually require complex preprocessing and post-processing steps. However, advancements in deep learning, especially in the CNN model, have yielded favorable results for intelligent diagnosis based on heart sounds. The intelligent heart sound auscultation process includes signal acquisition, signal preprocessing, heart sound feature extraction, and model training [79]. Fernando [80] proposed a heart sound segmentation method based on the combination of RNN with attention mechanism, which can effectively learn features from irregular and noisy heart sounds. Liu [81] used gradient-enhanced DT, SVM, CNN, and residual convolutional recurrent networks to analyze heart sound signals. The results showed that the residual convolutional recurrent network model has the highest recognition accuracy and sensitivity for the four types of coronary heart disease heart sounds.

Pulse diagnosis is one of the most important diagnostic methods in TCM. Doctors use three fingers to touch the wrist at three specific positions, namely, inch, off, and ruler, to examine the pulse and determine the health of patients. With advancements in sensors, detectors, and sensor technologies, digital palpation data can now be obtained from the same location, enabling AI technology to process palpation data and make diagnoses [82]. At present, most of the artificial intelligence technologies used in pulse diagnosis are limited to classical machine learning algorithms and their improved versions, including SVM, RF, DT, and neural network. Each learning algorithm shows unique advantages in pulse diagnosis learning classification. SVM usually achieves better performance than other traditional algorithms [82, 83]. As a widely used and adaptable deep learning method, CNN model algorithms have been proposed for TCM pulse diagnosis. CNN is good at mining local features and classifying and extracting global features. Moreover, CNN has been shown to perform better than traditional methods in AI-assisted pulse diagnosis, with accuracy above 90% [71].

### Applications of machine learning in disease treatment and effect evaluation

Machine learning has recently been successfully applied in disease treatment and effect evaluation of TCM, such as in prescription recommendation, transition prediction, and treatment prognosis (Table 2). The treatment prognosis model has received increasing attention in the context of clinical diagnosis and treatment decision-making [84]. Zhang [85] utilized transformer and GAN to develop an auxiliary tool to prescribe TCM prescriptions based on the patient’s clinical electronic health records. In their approach, transformer was used for TCM prescription generation, while the GAN model aims to augment the training set to further enhance the overall system performance by reducing overfitting effect. Dong [86] proposed a TCM prescription recommendation based on subnetwork term mapping and deep learning. They used TCM clinical case data to construct a natural product-symptom-related knowledge graph, constructed a symptom network by combining a meta path method and knowledge graph, proposed a subnetwork-based symptom term mapping method, utilized CNN as the train model, and finally output the prediction probability of each natural product to obtain the recommended prescription [86]. Dengzhan Shengmai capsule is a patented TCM preparation for the secondary prevention of stroke. Lu [87] utilized SVM to classify the network matrix of the Dengzhan Shengmai capsule group at baseline versus after treatment. SVM classification revealed significant white matter network alterations after treatment in the drug groups, with an accuracy of 68.18%. Tang [84] used RF, SVM, logistic regression, and extreme gradient boosting to predict whether colorectal cancer recurrence and metastasis with TCM factors would occur within 3 years and 5 years after radical surgery. The results showed that the four methods all showed certain predictive ability (area under the curve values > 0.70). Liu [88] proposed a graph CNN model to predict formula efficacy. The performance of graph CNN for multi-classification of tonic formulae showed the best result in comparison with SVM, naive Bayes, logistic regression, DT, and K-nearest neighbor.

### Applications of machine learning in prediction of biomarkers in TCM

The continuous advancement of information technology and biotechnology has yielded substantial biomarker data for TCM investigations using machine learning. Zhang [89] used RF and least absolute shrinkage and selection operator (LASSO) regression to identify important characteristic genes of oxidative stress. The receiver operating characteristic results demonstrated that the model was better in prediction efficiency with an AUC of 0.873. They also found that Nobiletin, which targets PLA2G4, may indicate a third pathway for the treatment of acute myeloid leukemia. Zhang [90] utilized SVM and LASSO to screen the underlying feature biomarkers in four RNA microarray datasets of myocardial infarction. These two machine learning methods yielded 10 and 14 genes, respectively. IL1B and TLR2 were the intersection biomarkers obtained by SVM and LASSO. On the basis of these biomarkers, several natural products such as *dan shen* and *san qi*, were identified as the potential TCM preparations for the treatment of myocardial infarction. By utilizing machine learning (residual CNN and partial least squares discriminant analysis), fingerprint, and network pharmacology, Li [48] screened the potential biomarkers in different parts of *Wolfiporia cocos*. Yuan [91] used RF to construct a drug-target prediction model to predict the key targets of Corydalis Rhizoma in the treatment of cardiovascular and cerebrovascular diseases. Cong [92] utilized SVM, DT, and back-propagation neural network to predict novel and selective tumor necrosis factor-alpha converting enzyme inhibitors. In their work, the SVM model showed the best overall prediction accuracy (98.45%) (Table 2).

**Table 2:** Applications of machine learning in TCM research

Chinese medicine field	Correlation algorithm	Performance of the algorithm
Natural product development	Deep neurol network [47, 50], RF [53, 54, 58, 59, 93], SVM [51, 53, 54, 57, 59, 93], DT [59, 93], neural network [53, 59]	RF was better than SVM, neurol network and DT in screening hepatotoxic compounds [59]. RF model is more accurate than SVM and DT in identifying molecular characteristics of natural product compounds with the meridians of TCM [93]
Disease diagnosis	SVM [10, 61, 66, 81–83], DT [68, 81–83], neural network [45, 61–63, 65, 82, 83], RF [61, 64, 67, 82, 83], CNN [64, 67, 70–78, 81], RNN [80], LSTM [64], MLP [68], residual CNN [81]	Compared with SVM, DT, traditional neural network and RF, CNN achieves higher accuracy in tongue shape classification [71]; Residual CNN model with highest accuracy and sensitivity in sounds auscultation [81]; SVM usually achieves better performance than other traditional algorithms in pulse diagnosis [82, 83]; CNN perform better than traditional methods in pulse diagnosis, with accuracy above 90% [71]
Disease treatment and effect evaluation	SVM [84, 87, 88], RF [84], GAN [85], CNN [86, 88], DT [88]	CNN performs better compared with SVM and DT [88]
Prediction of biomarkers in TCM	RF [89], SVM [90, 92], Residual CNN [48], DT [92], neurol network [92]	SVM model is better (accuracy:98.45%) than DT, neurol network in predicting tumor necrosis factor-alpha converting enzyme inhibitors [92]

## Research foundation and future research direction

### Advantages of algorithm ensemble and establishment of syndrome element diagnosis model of coronary heart disease

Our team proposed that algorithm ensemble is more suitable for TCM data models. Although an algorithm model can realize the construction of a prediction model based on the data for certain characteristics, the lack of universality and transferability limits the applicability of such models in systematic research on the diagnosis and treatment process of symptom-syndrome-treatment-prescription-natural products. Therefore, considering the current status of artificial intelligence in TCM, unification of different diagnosis and treatment rules on the basis of syndrome elements through a combination of multiple algorithms may facilitate accurate calculation of the entire diagnosis and treatment process in symptom-syndrome-treatment-prescription-natural products. The general structure of an ensemble learning algorithm consists of a set of “individual learners”, which are used to create an ensemble using a certain policy [94]. Using the ensemble principle, the advantages of each model can be extracted to integrate the optimized fusion model. Voting and stacking are common strategies for algorithm ensembles. A voting ensemble is a simple and effective fusion method that can weight the prediction results of a single model to improve model diversity while ensuring performance. The stacking ensemble is a more powerful learning-based ensemble strategy that uses the initial dataset to train a “component learner” and then generates a new data set for training a “meta-learner”. The training results show that the prediction performance of the integrated model on the training set and the test set is generally better than that of the single model. Fu [95] combined CNN with traditional algorithm models to analyze tongue-coating properties and found that the performance of the integrated model was improved. On the basis of the voting and stacking strategy, Yang [96] performed rule integration-model fusion on three machine learning models of SVM, RF, and neural network, and achieved good performance. Ge [97] proposed an ensemble algorithm that integrated the attention mechanism and LSTM, and showed that this ensemble algorithm can effectively select salient locations with higher accuracy and less computation.

**Fig. 6:**
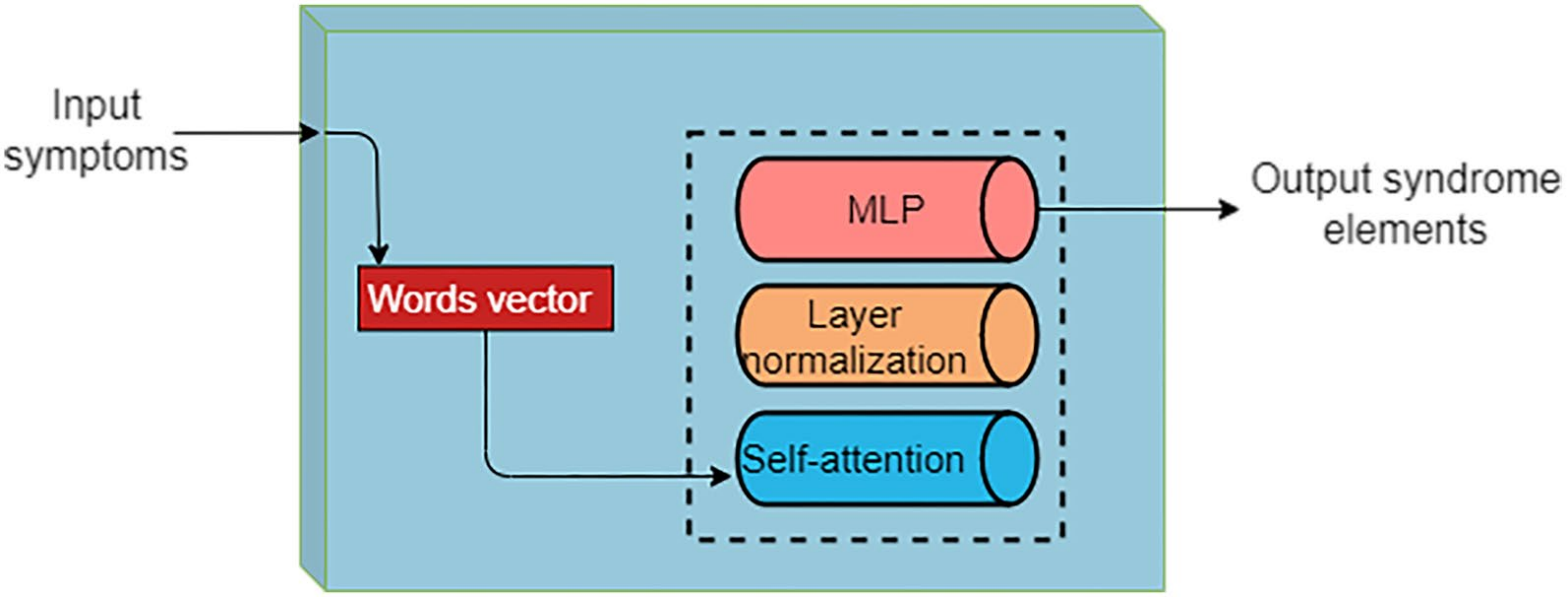
The diagnostic model of coronary heart disease syndrome elements by our team

The diagnosis and treatment theory in TCM shows the characteristics of diversified intersection. TCM treatment based on syndrome differentiation involves eight principal forms of differentiation, qi and blood fluid syndrome differentiation, zang-fu differentiation, and meridian syndrome differentiation. On the other hand, the compatibility rules include monarch and minister compatibility, flavor compatibility, and component compatibility. In addition, differences in the diagnosis and treatment rules among TCM sects and TCM physicians have made it difficult to reserve large high-quality TCM data of the same rule system. To address this problem, our team proposed syndrome elements that could link the symptoms, treatment, natural products, and prescriptions, and thereby unify different diagnosis and treatment rules [98]. The team used the improved transformer algorithm to construct a diagnostic model of coronary heart disease syndrome elements. This transformer model integrated the Seq2Seq module of RNN, LSTM and self-attention mechanism. At the same time, the multi-head attention mechanism, compound word vector, and random inactivation are used to study the syndrome elements of coronary heart disease (Fig. 6).

### Proposal of an application that combining machine learning with TCM theory and natural product computational biology

The effective chemical components of natural products have attracted attention worldwide. Rapid screening and identification of potential candidate compounds are very vital to determine the mechanisms underlying the therapeutic effects of drugs and can greatly ameliorate the development of new drugs. Since the successful development of artemisinin, the expectations for discovery of novel drugs with high efficacy and minimal adverse effects from TCM have increased. In this regard, a combination of the knowledge of effective chemical components of natural products with computational biology and machine learning under the optimization of TCM syndrome differentiation theory can facilitate disease treatment to realize effective development of machine learning in TCM clinical practice. The team proposed machine learning using a combination of computational biology of natural products and TCM theory. In this approach, computational biology is first used to study the pharmacology of natural products. Using the key molecular targets of disease as the research aspect, computational biology is used to simulate and screen the effective components of natural products for the key molecular target of disease. Based on molecular mechanisms identified in natural products screening, machine learning is performed on the selected natural products to establish a prediction model of molecular characteristics of natural products compounds and attributes, TCM syndrome differentiation, and meridians. For disease clinical data collection, the establishment of patient syndrome model in combination with a TCM theory screening model can allow optimization of the final therapeutic drugs.

The study used computational biology methods to analyze and screen natural products. Computational biology can unravel the seemingly impenetrable complexity of biological systems by an integrated approach which employed high-performance computers, state-of-the art software and algorithms, mathematical modeling, and statistical analyses [99]. Molecular dynamics, which is a branch of computational biology, can simulate the molecular mechanisms of effective chemical molecules of natural products acting on the body. Using this approach, the effective chemical molecules of natural products can be screened out to future identify natural products which are effective to remedy disease. Fang [100] summarized various cheminformatics, bioinformatics, and systems biology resources used to reconstruct drug-target networks for natural product medicine. Fu [101] developed a data-clustering method using a collection of 2,012 compounds associated with natural products and found that the cold and hot properties of natural products can be related to the physicochemical and target pathways of their constituent compounds. Wang [93] used DT, SVM, and RF algorithms for the first time to link the molecular characteristics of natural products compounds with the meridians of TCM. They identified the molecular characteristics of 646 natural products and their active constituents, including structure-based fingerprints and absorption, distribution, metabolism, and excretion characteristics. The meridian properties of TCM were predicted by machine learning methods, with the highest accuracy of 0.83, and RF showed the best accuracy.

**Fig. 7:**
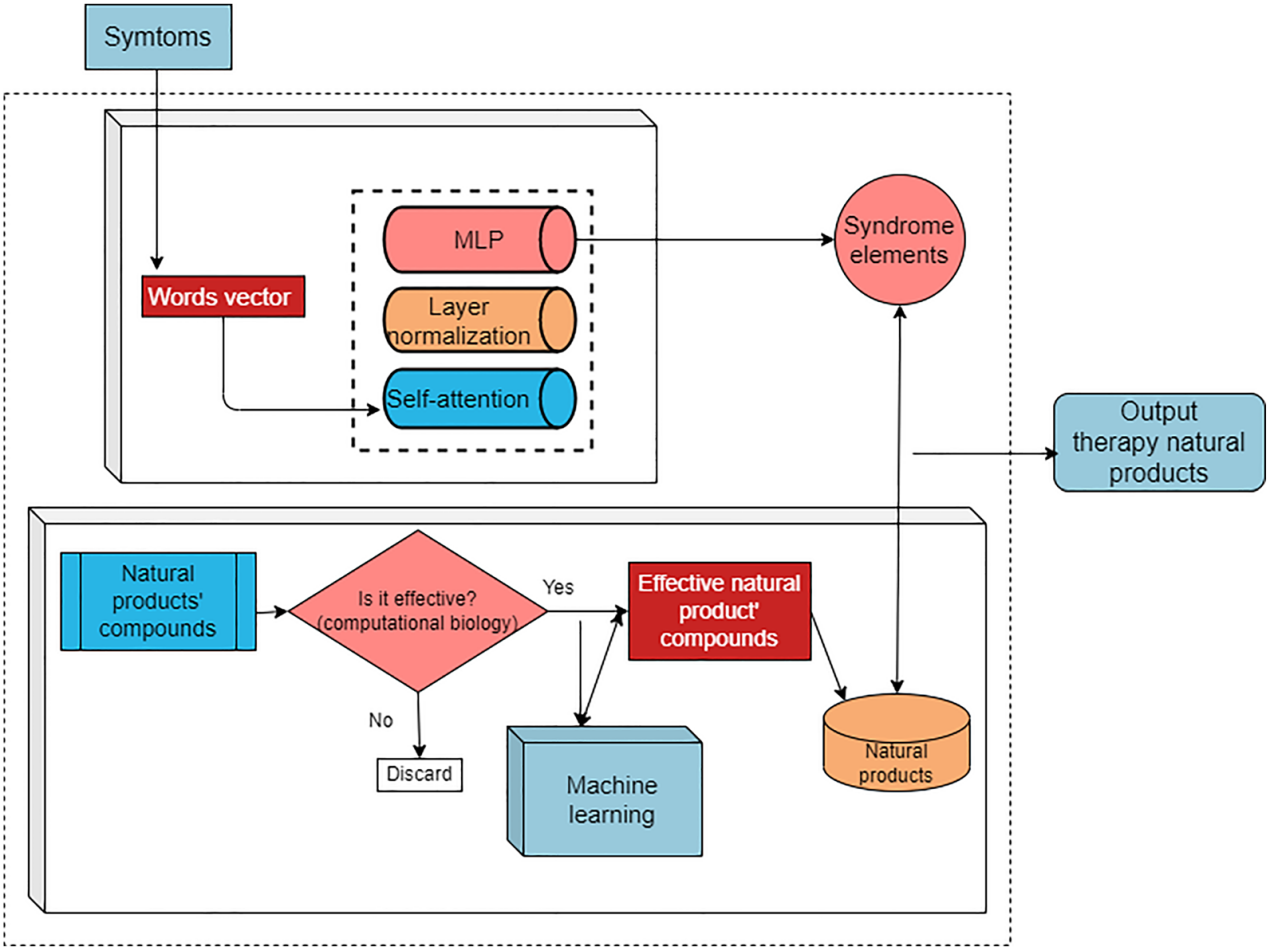
The perspective study of integrated methods including machine learning, TCM theory, natural product ingredients, and computational biology

Syndrome differentiation and treatment form the core of TCM theory. The development of intelligent diagnosis based on machine learning provides a dialectical basis for TCM syndrome differentiation. The use of machine learning in the research and development of TCM provides a basis for syndrome differentiation, while the unified diagnosis and treatment rules based on syndrome elements provide a direction for syndrome differentiation. Machine learning with molecular basis underlying syndrome elements may better classify diseases and improve clinical treatment effectiveness. Now, a web platform named SoFDA (http://www.tcmip.cn/Syndrome/front/#/) which is a network-based evaluation tool of multi-way associations among diseases, syndrome differentiation, and prescriptions could facilitate the understanding of syndrome differentiation and natural products from the perspective of molecular biology, enriched gene ontology terms or signaling pathways associated with syndrome differentiation [102]. Syndrome differentiation and treatment theory also conduct the relationship between natural products and syndrome elements which based on the function of natural products and their matched syndrome elements. By contacting natural products, syndrome elements, and molecules, promising research that combining machine learning with TCM theory and natural product computational biology were proposed. The stem diagram of these proposal is shown in Fig. 7. After using machine learning algorithms to intelligently diagnose disease and syndrome differentiation, the natural products screened by computational simulation can be used to realize intelligent diagnosis and treatment according to the results of intelligent differentiation of TCM. This process will use computational biology, machine learning, and TCM theory to achieve intelligent diagnosis and treatment of TCM, and is a potential research direction for TCM machine learning.

## Conclusion

In summary, this study reviewed the applications of machine learning in TCM research, including the principles of deep learning and traditional machine learning algorithms, the application of machine learning algorithms in TCM research, and an analysis of promising research directions. Machine learning has been applied in natural product research, TCM disease diagnosis, disease treatment and effect evaluation, and prediction of biomarkers in TCM. Traditional machine learning algorithms such as SVM, RF, DT and MLP are widely used in TCM learning. With advancements in machine learning, deep learning has found additional applications in TCM. Deep learning shows higher prediction performance than traditional machine learning algorithms. Although the clinical diagnosis and treatment process in TCM produces large amounts of data, the diversity in diagnosis and treatment models has resulted in a lack of uniform standards. Thus, the use of syndrome elements as a unified standard is important for addressing the difficulties in developing artificial intelligence-based techniques for TCM. The multi-algorithm rule integration proposed herein is more suitable for a TCM data model. Natural products contain many chemical components that influence the therapeutic effects. By utilizing computational simulation, these medicines can be screened at the molecule level. TCM theory serves as the guideline for the use of natural products, and the integration of machine learning with TCM theory, natural product, and computational simulation can yield an intelligent artificial intelligence-driven diagnosis and treatment model based on the effective chemical components of natural products under the guidance of TCM theory. Thus, the combination of machine learning with our understanding of effective chemical components of TCM and TCM theory offers a practical direction for the use of artificial intelligence in TCM, which can be expected to have far-reaching implications.

Although the development of machine learning in TCM is a promising study, the challenges and difficulties cannot be ignored. Effectiveness and safety are issues that need to be paid attention to in the development of artificial intelligence in TCM. The accurate application of machine learning in TCM theory which is the guiding program of TCM is related to the clinical effectiveness of TCM artificial intelligence research, and is a difficulty and challenge that TCM artificial intelligence needs to solve. The clinical effectiveness of intelligent diagnosis and treatment of the chemical molecular mechanism of natural products under the guidance of correct TCM theory is a worth working. Safety in TCM intelligent diagnosis and treatment is another key point that influence its development. The potential for drug-induced injury should be taken into account in TCM artificial intelligence research.

## Data Availability

Not applicable.
